# Transplant ureteral anastomosis to an inflatable penile prosthesis reservoir: A unique complication of renal transplantation

**DOI:** 10.1016/j.eucr.2026.103665

**Published:** 2026-11-03

**Authors:** Tessa Childers, Robert Helm, Paula Domino

**Affiliations:** a School of Medicine, University of Mississippi Medical Center, 2500 N. State Street, Jackson, MS, 39216, USA; b Department of Surgery, Division of Urology, 2500 N. State Street, Jackson, MS, 39216, USA

**Keywords:** Renal transplantation, Penile prosthesis, Ureteral anastomosis, Ureteroneocystostomy

## Abstract

We report a rare case of inadvertent anastomosis of transplanted ureters to the reservoir of a three-piece inflatable penile prosthesis (IPP) following renal transplantation. Four days postoperatively, the patient presented with suprapubic tenderness, left hemiscrotal swelling, and decreased urine output. Ultrasonography and computed tomography demonstrated ureteral stents terminating within the IPP reservoir. Exploratory laparotomy with IPP removal, malleable prosthesis placement, ureteral de-implantation, and ureteroneocystostomy was performed. This case highlights the importance of identifying an existing IPP when operating on nearby structures to prevent graft-threatening complications. Temporary malleable penile prosthesis placement appears to be a safe alternative when infection is absent.

## 1 Introduction

Renal transplantation is a widely performed procedure for end-stage renal disease, with approximately 27,500 renal transplants performed in 2025.^1^ Common postoperative complications include ureteral stricture, urinary leakage, urinary retention, lymphocele formation, postoperative hemorrhage, and renal vein thrombosis.^2^, ^3^ Erectile dysfunction (ED) affects up to 30 million men in the United States.^4^ According to American Urological Association guidelines, IPP placement is an appropriate option for men with ED, Peyronie's disease, refractory priapism, or untreated priapism lasting longer than 36 hours.^4^, ^5^ It is estimated that approximately 20,000 IPP placements are performed each year.^6^ Removal of an implanted IPP is most commonly due to infection, device malfunction, or corporal erosion.^7^ We report a unique case in which the IPP was removed following inadvertent anastomosis of the transplanted ureters to the reservoir of a previously placed three-piece IPP in a patient undergoing a two kidney en bloc allograft transplantation.

## 2 Case presentation

The patient is a 36-year-old male with a history of end-stage renal disease secondary to hypertensive nephrosclerosis, sickle cell trait, and ED. The patient's surgical history included placement of a three-piece IPP performed without complication, but no other pelvic surgeries. The reservoir was placed in the right lower quadrant anterolateral to the urinary bladder within the space of Retzius. Eighteen months post IPP placement, the patient underwent a deceased donor renal transplant in the right lower quadrant. Two kidneys were transplanted en bloc with bilateral ureteral stent placement during an uneventful surgery performed by the transplant team. The Foley catheter was removed on postoperative day three to reduce the risk of erosion of the penile prosthesis. Urology was consulted on postoperative day four for oliguria and acute kidney injury.

The patient reported suprapubic tenderness and increased swelling and tenderness of the left hemiscrotum following renal transplantation surgery. The IPP had last been cycled several days prior to surgery, and the patient did not report new device issues. On physical exam, the penis was partially erect, with bilateral IPP cylinders in appropriate position without evidence of erosion. Moderate scrotal edema without erythema was noted. The patient's preoperative serum creatinine was 9.71 mg/dL and increased to 11.70 mg/dL, 11.57 mg/dL, and 12.08 mg/dL on post operative days two, three, and four respectively.

Ultrasonography was performed to evaluate the renal transplant graft due to oliguria with acute kidney injury [Fig. 1]. Computed tomography revealed that the transplanted ureters were anastomosed to the IPP reservoir with ureteral stents terminating within the IPP reservoir in both axial [Fig. 2] and coronal views [Fig. 3]. This resulted in postoperative oliguria and acute kidney injury due to diversion of urine into the closed prosthetic system. Cystoscopy and cystogram demonstrated no communication between the implant and bladder. The patient subsequently underwent an expedited exploratory laparotomy with removal of IPP components followed by ureteral de-implantation from the IPP reservoir, removal of the reservoir, and revision with ureteroneocystostomy. Intraoperatively, the IPP reservoir, pump, and cylinders showed no signs of infection. Therefore, we elected to replace the inflatable device with a malleable prosthesis to maintain corporal patency. There was no purulence noted on the field or other signs of infection.

**Fig. 1:**
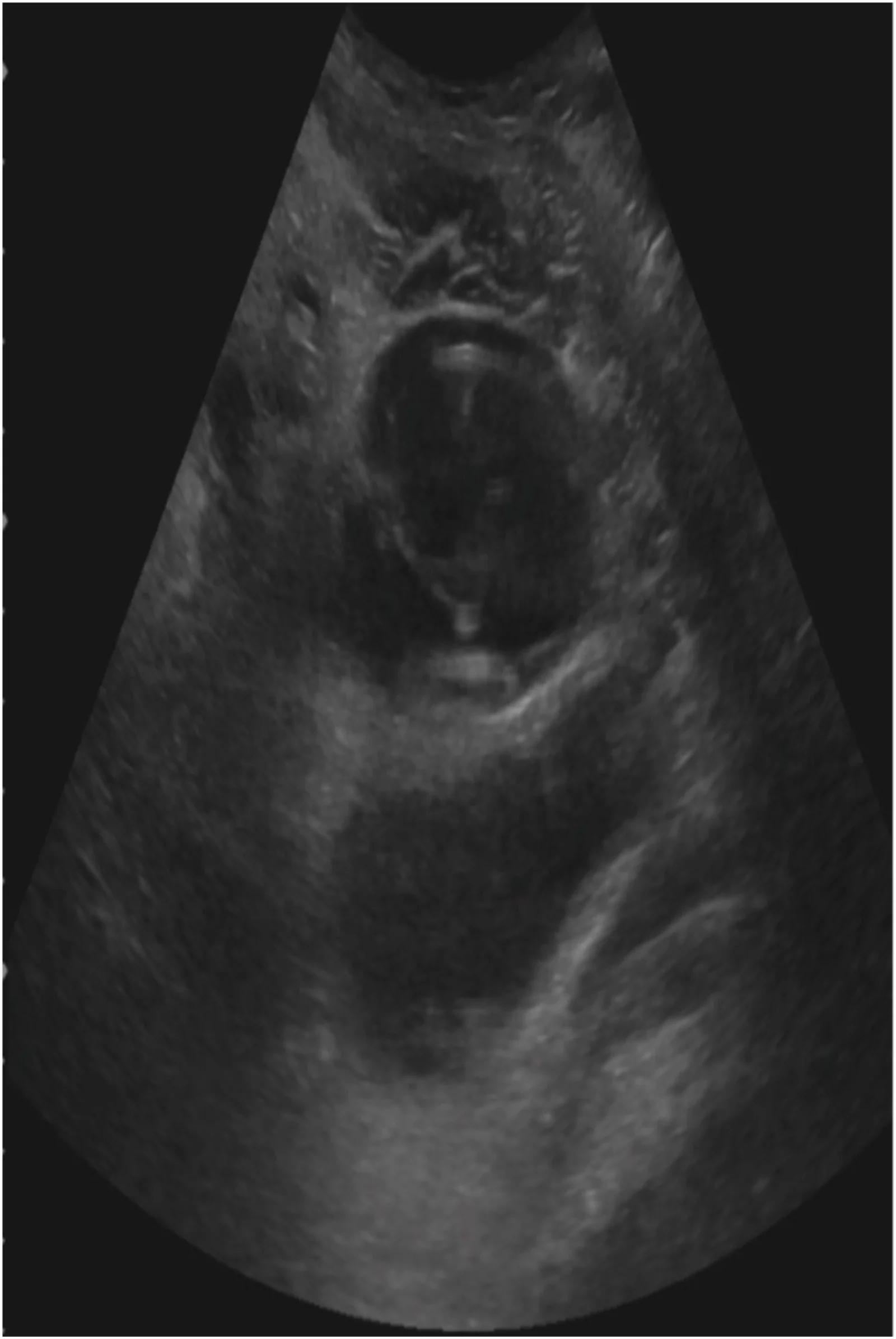
Transverse ultrasound of the urinary bladder and IPP reservoir.

**Fig. 2:**
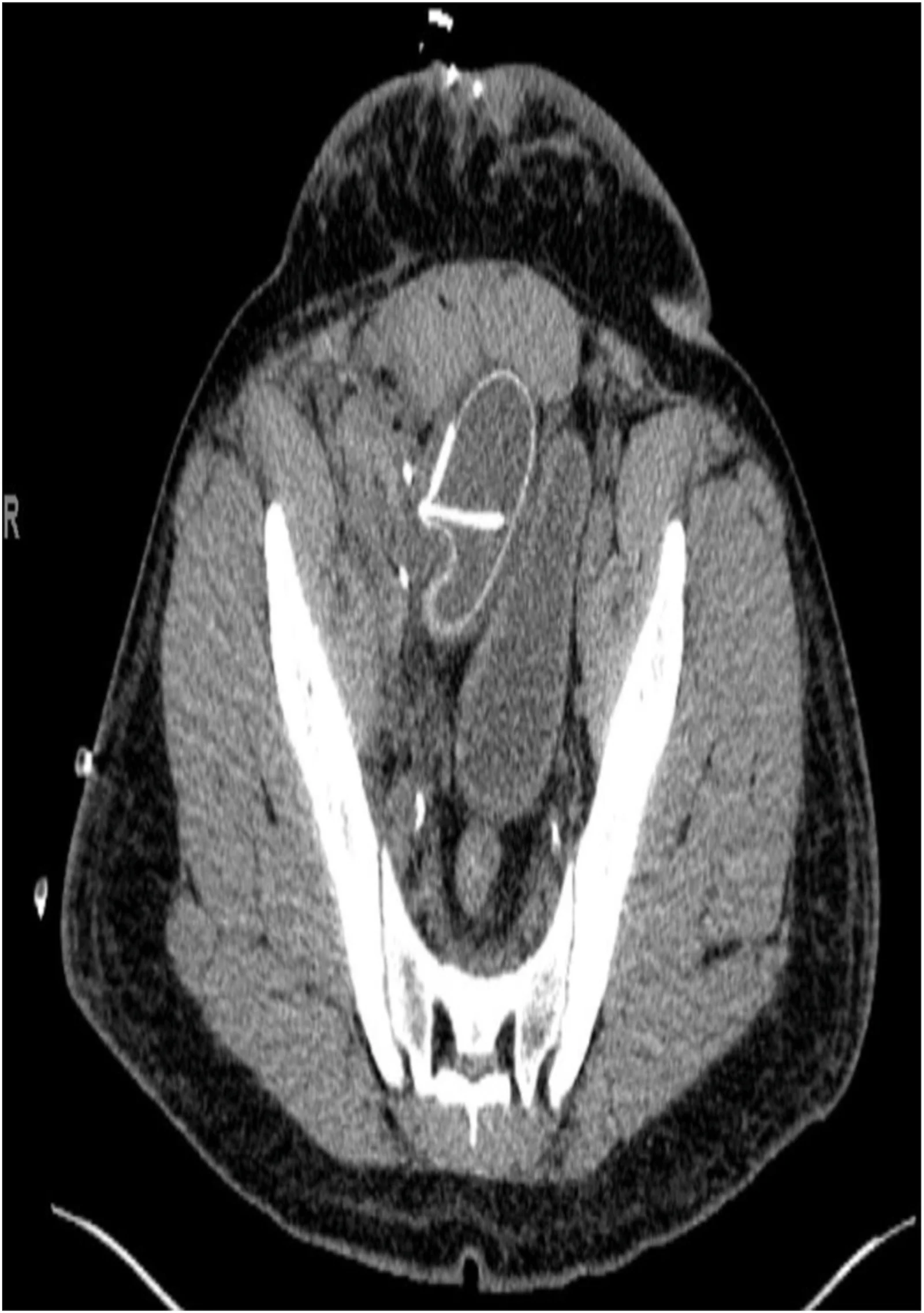
Axial computed tomography of the transplanted ureters anastomosed to the IPP reservoir with stents into the reservoir.

**Fig. 3:**
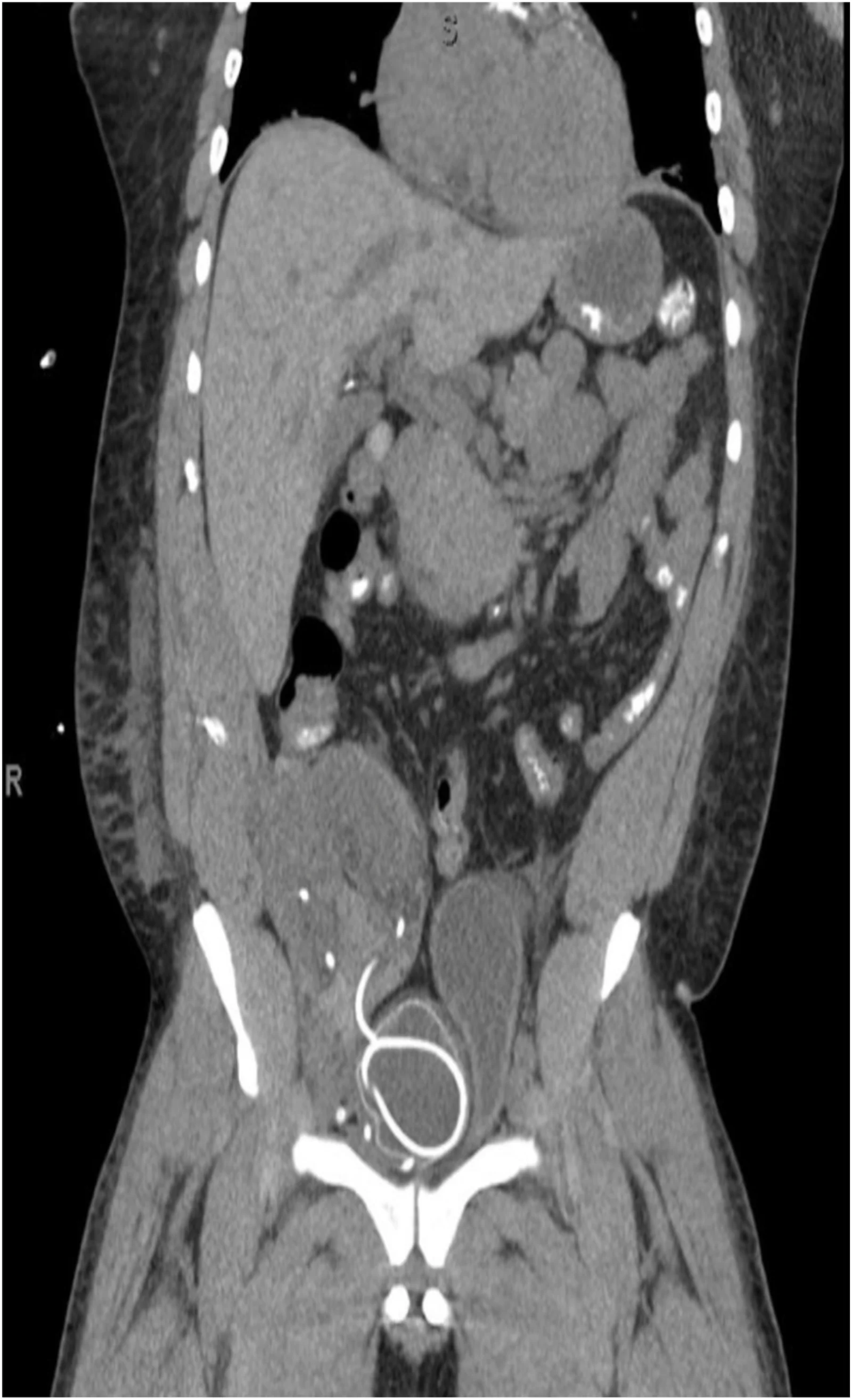
Coronal computed tomography of the transplanted ureters anastomosed to the IPP reservoir, showing ureteral stents into the reservoir.

Six months postoperatively, the patient reported dissatisfaction with the firmness of his erections with the malleable prosthesis. Given his complex abdominal surgical history and prior complications, he was counseled on and agreed to replacing the malleable prosthesis with a two-piece inflatable penile prosthesis. This was performed without complication. At a six-week postoperative appointment, the IPP was cycled and demonstrated appropriate function. The intracorporeal cylinders were well positioned with the tips at the mid-glans, the penis was straight, and there was no evidence of erosion. The penoscrotal incision was healing well without signs of infection. The patient reported no complaints and was pleased with both the cosmetic and functional results. The patient continues to follow with the transplant team and reports no issues.

## 3 Discussion

Many treatment options exist for ED, most of which provide temporary restoration of sexual function with ongoing treatment. Surgical options for erectile dysfunction include malleable (non-inflatable) implants, as well as two-piece and three-piece IPPs. Among these options, three-piece IPP placement is the most performed procedure.^8^ The device consists of two inflatable cylinders that are placed into the bilateral corpora cavernosa, a saline-filled reservoir positioned within the pelvis, and a scrotal pump. Three-piece IPPs are associated with the highest satisfaction rates, with patient and partner satisfaction rates of 75-98% and 85.4%, respectively.^9^ Despite the high satisfaction rates, this treatment option remains an invasive procedure, with infection of the prosthesis being the most significant postoperative complication. Infectious complications occur in 1-3% of primary prosthesis placement procedures and 8-18% of prosthesis revisions.^10^ Noninfectious postoperative complications related to the prosthesis reservoir, such as reservoir rupture and mechanical malfunction, are relatively uncommon.^10^

In the setting of postoperative infectious complications, surgical removal of the prosthesis is indicated with reimplantation months later.^11^ Alternatively, there is literature supporting the salvage technique, which involves placement of a new penile prosthesis immediately after the removal of an infected prosthesis.^12^ For postoperative noninfectious complications, the literature supports complete removal of the penile prosthesis.^13^ This recommendation applies even when a single component of the prosthesis is compromising device performance. There are higher rates of infectious and noninfectious postoperative complications after undergoing partial component revision rather than complete prosthesis revision.^13^

Regarding our case, the transplanted ureters were anastomosed to the patient's penile prosthesis reservoir overlying the right anterior bladder wall. This resulted in diversion of urine into the closed system of the device, disrupting prosthesis function, increasing the risk of infection, and compromising graft function. After review of the index operative report, it did not describe any abnormalities or difficulties during the case. Per our literature review, there are no other reported cases of this specific postoperative transplant complication; therefore, there were no guidelines to consider. Studies show that implant revision procedures are at higher risk of infection than primary implantations in all patients, but published literature does not demonstrate an increased risk of IPP infection in solid organ transplant recipients.^10^, ^14^ In our case, we chose to remove all components of the prosthesis, which revealed no signs of infection of the reservoir, pump, or cylinders. A malleable penile prosthesis was placed following complete removal. The field was thoroughly irrigated with antimicrobial solution throughout the explantation and implantation of the devices. After his six-month follow-up, the patient denied any complications but reported dissatisfaction with the firmness of his erections with the malleable prosthesis. Given his complex past abdominal surgical history, the patient elected to replace the malleable prosthesis with a two-piece inflatable penile prosthesis. Six weeks after placement, the two-piece IPP cycled well, the intracorporeal cylinders were appropriately positioned, and there was no evidence of infection. The patient had a good erectile result and reported no complaints.

## 4 Conclusion

Inadvertent ureter-to-IPP reservoir anastomosis is a rare but important complication of renal transplantation in patients with prior IPP placement. When operating on patients with an IPP, identification of the anatomical location of the IPP components is critical to prevent serious postoperative complications. If such complications arise, prompt recognition of symptoms, early diagnostic imaging, and timely intervention are critical to optimize patient outcomes. Management requires emergent reimplantation of the transplanted ureters to preserve graft function, followed by appropriate management of the IPP. In the absence of infection, replacement of the inflatable device with a malleable prosthesis appears to be a safe and feasible strategy.

## CRediT authorship contribution statement

**Tessa Childers:** Writing – review & editing. **Robert Helm:** Writing – original draft. **Paula Domino:** Supervision.

## Ethics declaration

Written informed consent to take part in the study and to publish the article has been obtained from all participants or their legal representatives. The privacy rights of participants have been observed.

This study was performed in compliance with relevant laws, regulatory frameworks and guidelines where the research took place.Ethics committee approval was not required under relevant laws and institutional guidelines. Institutional review board approval was not required for this single-patient case report in accordance with institutional policy.

This research follows the CARE guidelines and the CARE checklist.
